# Occurrence and Molecular Characterization of Potentially Pathogenic *Vibrio* spp. in Seafood Collected in Sicily

**DOI:** 10.3390/microorganisms11010053

**Published:** 2022-12-23

**Authors:** Annamaria Castello, Vincenzina Alio, Sonia Sciortino, Giuseppa Oliveri, Cinzia Cardamone, Gaspare Butera, Antonella Costa

**Affiliations:** Food Microbiology Sector, Istituto Zooprofilattico Sperimentale della Sicilia “A. Mirri”, 90129 Palermo, Italy

**Keywords:** pathogenic *Vibrio* spp., antimicrobial resistance, molecular characterization, food safety, seafood

## Abstract

Seafood can vehiculate foodborne illnesses from water to humans. Climate changes, increasing water contamination and coastlines anthropization, favor the global spread of *Vibrio* spp. and the occurrence of antibiotic-resistant isolates. The aim of this study was to evaluate the spread of potentially pathogenic *Vibrio* spp. in fishery products collected in Sicily and to assess their antibiotic resistance. Bacteriological and molecular methods were applied to 603 seafood samples to detect *V. parahaemolyticus*, *V. cholerae*, *V. vulnificus*, and *Vibrio alginolyticus* in order to assess their pathogenicity and antimicrobial resistance. About 30% of bivalves and 20% of other fishery products were contaminated by *Vibrio* spp.; *V. parahaemolyticus* accounted for 43/165 isolates, 3 of which were carrying either *tdh* or *trh*; *V. cholerae* accounted for 12/165 isolates, all of them non-O1 non-O139 and none carrying virulence genes; and *V. vulnificus* accounted for 5/165 isolates. The highest rates of resistance were observed for ampicillin, but we also detected strains resistant to antibiotics currently included among the most efficient against *Vibrio* spp. In spite of their current low incidence, their rise might pose further issues in treating infections; hence, these results stress the need for a continuous monitoring of antimicrobial resistance among fishery products and an effective risk assessment.

## 1. Introduction

Seafood can be responsible for various foodborne illnesses due to the contamination of water by chemicals, metals, toxins, and infectious agents such as bacteria, viruses, and parasites [1]. Mussels in particular can filter a great amount of water; hence, they accumulate in their bodies various toxins and microorganisms from the environment, and their consumption can expose a high risk of food poisoning, especially when ingested raw or undercooked [2]. *Vibrio* species are autochthonous to marine, riverine, and estuarine environments; globally widespread in freshwater [3,4]; and commonly detected in seafood. To date more than 100 species of vibrios have been described, 13 of which are classified as human pathogens [5], even though with differences in terms of epidemiological relevance. *V. parahaemolyticus*, *V. cholerae*, and *V. vulnificus* are the most important pathogens for humans [6], and they are considered as a serious and expanding threat for public health. Vibriosis is also one of the main bacterial diseases of larvae and juvenile bivalves affecting the early development stages of shellfish growth, with deleterious effects on both the aquaculture sector and aquatic ecosystems.

*V. parahaemolyticus* occurs naturally in the marine environments and may be abundant in shellfish. It is considered one of the leading causes of seafood-borne diseases [7] and recognized as a common cause of acute gastroenteritis worldwide [8]. Moreover this is considered an emerging species because of its involvement in outbreaks subsequent to the consumption of contaminated food, in particular undercooked fish and shellfish [9,10]. Among all strains of *V. parahaemolyticus*, the ones responsible for the majority of disease symptoms and deaths are characterized by two virulence genes associated with enteropathogenicity: a thermostable direct hemolysin (TDH) and a thermostable related hemolysin (TRH), encoded by the *tdh* and *trh* genes respectively [11]. So far only a few foodborne outbreaks from *V. parahaemolyticus* or sporadic cases have been reported in Europe [12,13], while many foodborne outbreaks have been reported in the United States, Chile, and Japan [10].

*V. cholerae* is a cosmopolitan aquatic species whose ability to survive in different environmental niches is attributed to its adaptation to nutrient deprivation, fluctuations in salinity, and temperature [10,14]. Strains within the serogroups O1 and O139 produce cholera toxin and are the causative agents of endemic and epidemic cholera, representing an important cause of morbidity and mortality in countries with inadequate access to clean water and sanitation facilities [15]. In 1994, cholera outbreaks occurred also in Italy and Albania [3,16]. The cholera disease requires quarantine and must be reported to the World Health Organization. *V. cholerae* strains not included in the aforementioned serogroups as well as other *Vibrio* spp. are referred to as non-cholera *Vibrio* spp. and have a worldwide distribution, especially in warm estuarine and marine ecosystems [17]. The number of outbreaks caused by *V. cholerae* non-O1/O139 is increasing over time, probably also because of the progressive rise of sea surface temperature [18,19]. Europe lacks mandatory notification systems for *Vibrio*-associated illnesses other than those caused by *V. cholerae* O1/O139, and this prevents an accurate estimation of the number of infections.

*V. vulnificus* is an opportunistic human pathogen responsible for 95% of seafood-related deaths in the USA [20]. *V. vulnificus* biogroup 1 infects humans through the ingestion of contaminated seafood or skin lesions. Following infection, healthy individuals may suffer from gastroenteritis or wound infections, whereas in immunocompromised hosts the infection often leads to primary or secondary septicemia, with a high mortality rate [21].

*V. alginolyticus* is ubiquitous in marine and estuarine environments, and people can come into contact with this bacterium through water exposure or through eating contaminated seafood [22]. Nevertheless, *V. alginolyticus* is more frequently related to ear infections rather than foodborne illnesses, and it has been mostly reported as the cause of wound infections through the exposure of cuts or abrasions of the skin to seawater containing *Vibrio* [23] and among the most frequent causative agents for nonfoodborne *Vibrio* infections (NFVI) in the USA [24]. Even though *V. alginolyticus* is not included among the most relevant foodborne pathogens, this species is particularly widespread in the aquatic environment and may play an important role as a reservoir of resistance genes, favoring their diffusion among other pathogenic species possibly present in the same microbial communities.

A rapidly warming marine environment, together with the increase of extreme weather events such as heatwaves, is supporting the spread of *Vibrio* spp. Worldwide, and larger *Vibrio* spp. outbreaks have been reported recently in temperate regions such as Spain [25], Sweden, and Finland [13]. Moreover, resistance to various antibiotics has emerged over the last decades among the *Vibrio* spp. circulating in marine environments [26,27]. The acquisition of resistance in bacteria or bacterial communities arises via genetic transfer events under the positive pressure of various phenomena currently on the rise, such as the environmental antimicrobial contamination [28,29] and the emergence of microenvironments with high-density microbial communities like highly anthropized aquatic environments or sewage treatment plants, where indigenous bacteria and those derived from humans and animals co-exist [30]. Naturally occurring aquatic bacteria, including pathogenic *Vibrio* strains, can serve as a reservoir of resistance genes and may play an important role in the evolution and spread of antibiotic resistance in aquatic environments [31,32,33,34]. Considering that seafood and especially bivalves can concentrate these bacteria, they also represent a potential reservoir of resistance genes, transmitted to humans through their consumption. For this reason, monitoring the occurrence of antibiotic resistance among *Vibrio* spp. isolated from these samples is of great relevance for a deeper assessment of human exposure to foodborne risks, as well as a better estimation of the anthropic impact on local environments and data collection for improving control strategies. Referring to the evaluation of prevalence and genetic characterization of potentially pathogenic vibrios in seafood, while data are available from investigations conducted in various Italian regions, including the south and Sardinia [2,3,35,36], with only limited data reported from Sicily. The present study aims to provide a contribution to the knowledge of the occurrence and potential pathogenicity of *V. parahaemolyticus*, *V. cholerae*, and *V. vulnificus* in shellfish and fishery products collected in Sicily, as well as on antibiotic resistance profiles of the circulating strains.

## 2. Materials and Methods

Sampling was carried out from January 2015 to December 2019 in retail outlets and shellfish farms. The analyses described were performed on a total number of 603 seafood samples, including 366 mussels, 46 clamps, 18 oysters, 139 fish, 28 cephalopods, and 6 crustaceans. All samples were maintained at +4 °C and processed immediately after their arrival at the laboratory. Shellfish were rinsed in sterile distilled water to remove any debris on the shell, then opened aseptically in accordance with UNI EN ISO 6887-3/2003 standard procedure. The content of each bivalve (flesh and intravalvular liquid) was transferred into stomacher bags up to a total weight of 25 g per each sample, then mashed in a stomacher for 2 min. Microbiological analyses were performed on the homogenate.

Referring to fish, cephalopods, and crustaceans, 25 g of tissues were split aseptically from each sample and transferred into stomacher bags for the subsequent procedures.

### 2.1. Isolation and Identification of Vibrio *spp.*

A pre-enrichment was performed by diluting 25 g of each sample in 225 mL of alkaline peptone water (APW) and incubating at 37/41.5 °C overnight. At the end of the incubation period, an inoculating loop of the culture broth was seeded onto thiosulfate citrate bile salt agar (TCBS) (Microbiol, Cagliari, Italy) and CHROMagar *Vibrio* (CHROMagar™, Paris, France) plates. Plates were incubated for 24 h at 37 °C. After incubation, presumptive colonies from *V. parahaemolyticus* (green on TCBS Agar, mauve on CHROMagar), *V. cholerae* (yellow on TCBS Agar, turquoise on CHROMagar), and *V. vulnificus* (green on TCBS Agar, turquoise on CHROMagar) were streaked onto trypticase soy agar (TSA) supplemented with 3% NaCl, and their phenotypes were characterized by the following laboratory protocol: oxidase and catalase tests, Gram staining, sugar fermentation, and sensitivity to vibriostatic agent O12. Biochemical identification was performed following the API 20NE identification system (BioMerieux, Marcy l’Etoile, France). Also the strains identified as *V. cholerae* were processed by agglutination tests, using commercial polyvalent anti-O1 and anti-O139 antisera (Denka Seiken Co., Ltd., Tokyo, Japan). All isolates presumptively identified as *V. parahaemolyticus*, *V. cholerae*, and *V. vulnificus* were confirmed and further characterized by means of molecular tests.

### 2.2. Molecular Analyses

All isolates biochemically identified as *V. parahaemolyticus* were confirmed by PCR for the detection of a highly conserved species-specific marker gene (*toxR*) [37]. Virulotyping of the isolates identified as *V. parahaemolyticus* was performed with a PCR assay targeting the virulence genes associated with enteropathogenicity (*tdh* and *trh*) according to the protocols described by Bej et al. [38]. Appropriate primer sets targeting the three aforementioned genes were designed, and each batch of PCR assays included *V. parahaemolyticus* NTCT 10884-ATCC 17802 as a positive control and molecular grade water as negative control. All isolates biochemically identified as *V. vulnificus* and *V. cholerae* were confirmed by PCR for the detection of species-specific marker genes (*VVH* and *prVC*, respectively). Each batch of PCR assays included *V. vulnificus* ATCC 27562 and *V. cholerae* ATCC 1473A as positive controls and molecular grade water as negative control. The primer sequences and the expected sizes for each amplicon are listed in Table 1.

Bacterial DNA was extracted using the following protocol: an isolated colony from saline nutrient agar was resuspended in 1 mL physiological solution or ultrapure water. Each tube was boiled for 5 min at 95 °C, cooled at 4 °C, and centrifuged at 10,000× *g* for 1 min. The supernatant was collected for DNA amplification. PCR assays targeting *ToxR* and *VVH* loci were performed under the following conditions: a first step denaturation at 96 °C for 5 min and 30 cycles of a 3-step amplification (denaturation at 94 °C for 1 min, annealing at 63 °C for 1.5 min, and extension at 72 °C for 1.5 min), followed by a final extension step at 72 °C for 7 min. PCR assays targeting the VC locus were performed under the following conditions: a first step denaturation at 94 °C for 2 min and 30 cycles of a 3-step amplification (denaturation at 94 °C for 1 min, annealing at 50 °C for 1 min, and extension at 72 °C for 1.5 min) followed by a final extension step at 72 °C for 10 min. PCR assays targeting *tdh* and *trh* loci were performed under the following conditions: a first step denaturation at 94 °C for 3 min and 30 cycles of a 3-step amplification (denaturation at 94 °C for 1 min, annealing at 58 °C for 1 min, and extension at 72 °C for 1 min) followed by a final extension step at 72 °C for 5 min. After PCR amplification, 10 μL of each reaction product were loaded into 2.0% agarose gels in Tris-acetate-EDTA buffer (Bio-Rad Laboratories, Hercules, CA, USA), containing 5 µL SYBR^®^ Safe DNA gel stain (Thermo Fisher Scientific, Waltham, MA, USA). The amplicons were visualized under a UV transilluminator (GelDoc-It Imaging System, EuroClone, Milano, Italy). The isolates of non-O1 non-O139 *V. cholerae* (NCV) were processed to detect the virulence genes *ctxA, stn*/*sto* (non-O1 heat-stable enterotoxin)*, tcpA*, and *hlyAET* [40].

### 2.3. Antimicrobial Susceptibility

Antimicrobial susceptibility patterns were determined for 165 strains of *Vibrio* spp. Isolated from seafood that included 105 *V. alginolyticus isolates*, 43 *V. parahaemolyticus*, 12 *V*. *cholerae*, and 5 *V. vulnificus*. The aforementioned assessments were performed with the Kirby–Bauer method, using the following 15 antimicrobials: ampicillin (Amp; 10 μg), cefotaxime (Ctx; 30 μg), ceftriaxone (CRO; 30 µg), ceftazidime (Caz; 10 μg), cephalothin (Kf; 30 μg), ciprofloxacin (Cip; 5 μg), chloramphenicol (C; 10 μg), gentamicin (Cn; 10 μg), kanamycin (K; 30 μg), streptomycin (S; 10 μg), trimethoprim/sulfamethoxazole (Stx; 25 μg), tetracycline (Te; 10 μg), colistin sulphate (Ct; 10 μg) cefazolin (Kz; 30 µg), and amoxicillin/clavulanic acid (Amc; 20 μg/10 μg). Inhibition zones were measured and interpreted according to the guidelines of the Clinical and Laboratory Standards Institute (CLSI) [41].

### 2.4. Data Analysis

The prevalence of *Vibrio* was calculated with a 95% confidence interval (CI). Chi-square test was used for comparison of the prevalence, and the significance level was set at *p*-value = 0.01.

## 3. Results

### 3.1. Isolation and Identification of Vibrio *spp.*

**Figure 1:**
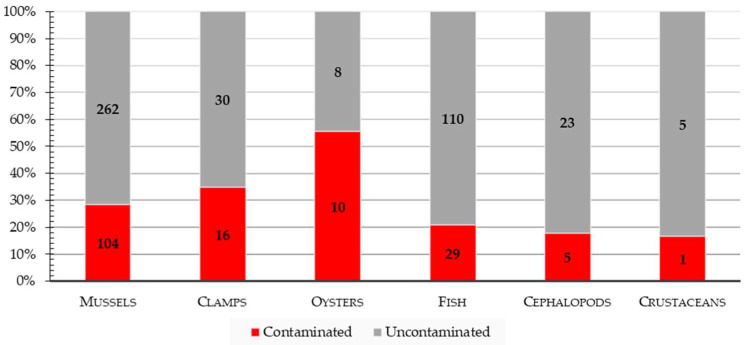
Bar graph showing the percentage of samples contaminated by *Vibrio* spp. Per each group enlisted.

Among the 603 samples analyzed, 165 (27.4%) were contaminated with *Vibrio* spp. (95% CI: 23.8 to 30.9%). Those included 104/366 (28.4%) samples of mussels, 16/46 (34.8%) samples of clamps, 10/18 (55.5%) samples of oysters, 29/139 (20.9%) samples of fish, 5/28 (17.8%) samples of cephalopods, and 1/6 (16.7%) samples of crustaceans (Figure 1). The prevalence of *Vibrio* spp. was significantly higher in oysters compared to fish and cephalopods (*p* < 0.01), while the slightly higher prevalence revealed in comparison to other species of bivalves and crustaceans was not statistically significant.

**Figure 2:**
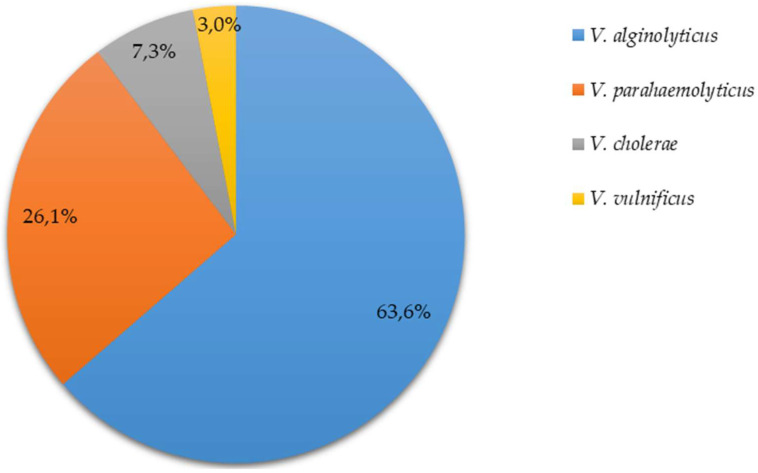
Pie graph depicting the percentage of contaminated samples that tested positive for *V. parahaemolyticus*, *V. cholerae*, *V. vulnificus*, and *V. alginolyticus*.

Among the 165 *Vibrio* spp. Detected, 105 isolates (63.6%) were identified as *V. alginolyticus*, 43 isolates (26.1%) were identified as *V. parahaemolyticus*, 12 isolates (7.3%) were identified as *V. cholerae* (NCV), and 5 isolates (3%) were identified as *V. vulnificus* (Figure 2).

### 3.2. Molecular Analyses

PCR analyses revealed the virulence genes associated with enteropathogenicity (*tdh* and *trh*) in 3 out of 43 *V. parahaemolyticus* isolates (n = 2 mussels and n = 1 fish). In particular one isolate was *tdh*^+^/*trh*^−^ (2.3%), and two isolates were *tdh*^−^/*trh*^+^ (4.6%). Following serological analysis, the 12 strains of *V. cholerae* isolated were identified as non-O1 non-O139 *V. cholerae* (NCV). None of them carried the researched virulence genes (*ctxA*, *stn*/*sto*, *tcpA*, or *hlyAET*).

### 3.3. Antimicrobial Susceptibility

- *V. parahaemolyticus* isolates were resistant to ampicillin, cefazolin, cephalothin, streptomycin, and tetracycline at the rate of 70%, 30.2%, 21%, 19%, and 14%, respectively. Intermediate resistance was revealed to colistin sulphate (21%), ceftazidime and kanamycin (18.6%), and cefotaxime (14%). A pattern of multidrug resistance was exhibited in 2/43 isolates (Table 2). In contrast, all isolates were sensitive to four antimicrobials (ceftriaxone, ciprofloxacin, chloramphenicol, and trimethoprim/sulfamethoxazole), and >90% of the isolates were susceptible to amoxicillin-clavulanic acid and gentamicin.
- *V. cholerae* isolates were resistant to ampicillin (75%), trimethoprim/sulfamethoxazole (42%), ceftriaxone, ceftazidime, and cephalothin (17%). Intermediate resistance to cephalothin was shown in 50% of the isolates, and 17% exhibited intermediate resistance to streptomycin and ceftazidime. All isolates were susceptible to cefotaxime, ciprofloxacin, chloramphenicol, and tetracycline.
- All tested *V. vulnificus* isolates (n = 5) were susceptible to streptomycin, and a large percentage of strains were sensitive to all the tested antibiotics including drugs recommended by the Center for Disease Control and Prevention (CDC) for the treatment of *V. vulnificus* infections: tetracycline, ciprofloxacin, and trimethoprim/sulfamethoxazole. One strain, whereas, showed intermediate sensitivity to tetracycline and ciprofloxacin and resistance to cefazolin.
- Among the 105 *V. alginolyticus* isolates, 88.6% were resistant to ampicillin and 22% to gentamicin. Intermediate resistance to streptomycin and kanamycin were also observed, showing an incidence of 55.2% and 10.5%, respectively. All isolates were sensitive to the remaining antimicrobials.

## 4. Discussion

The aim of this study was to estimate the abundance of *Vibrio* spp. in Sicilian fishery products and to assess the prevalence of potentially pathogenic specimens. The higher prevalence of *V. parahaemolyticus* and the lower rate of confirmation for *V. cholerae* NCV and *V. vulnificus* are consistent with previous research carried out in Italy, also reporting the lower prevalence of the last two species compared to the first one [12]. In particular, a study carried out in two regions of Italy (Emilia Romagna and Sardinia) revealed a prevalence among clams (*Ruditapes philippinarum*) of 27.8% and 30.3% for *V. parahaemolyticus*, 10.1% and 6.1% for *V. vulnificus*, and 0% and 3% for *V. cholerae* in Emilia Romagna and Sardinia, respectively [3]. Less recently Normanno et al. [2] had reported a prevalence of 8% for *V. parahaemolyticus*, 3% for *V. vulnificus*, and 0.3% for *V. cholerae* among mussels (*Mytilus galloprovincialis*) sold in Puglia. As per our analyses, the majority of *Vibrio* spp. Isolates (105 out of 165) belonged to the species *V. alginolyticus,* which was the most abundant one, especially in mussels. This species can cause illness among both aquatic animals and humans, even though the mechanisms underpinning its pathogenicity are not fully understood. Moreover some strains may carry both virulence genes derived from pathogenic *V. cholerae* and *V. parahaemolyticus* [42] and antibiotic-resistant genes [43,44], thus acquiring increasing relevance under the “One-Health” perspective. In fact they can constitute a natural reservoir for virulence and antibiotic resistance genes that could reach other bacteria and new habitats, leading to new risks for humans and animals.

As reported previously by other authors, our results confirmed that potentially pathogenic *V. parahaemolyticus* constitutes a minority in this species. In fact Vongxay et al. [45] characterized *V. parahaemolyticus* pathogenicity in clinical and environmental samples, reporting that only 2% and 4% of the isolates carried the *tdh* gene and the *trh* gene, respectively. In addition, analogous analyses carried out on crustaceans collected in Italy revealed a 1.4% incidence of *tdh* gene among the pathogenic *Vibrios* detected [36]. Even though most of *V. parahaemolyticus* isolates are *tdh*/*trh*-negative, they should not be neglected. In fact, regardless of the *tdh* production, these bacteria can alter the epithelial barrier of the host, inducing rearrangements in the cytoskeleton, an apro-inflammatory response, and/or cell death due to the involvement of other virulence factors [46]. Consistently with these observations, various food poisoning outbreaks related to *V. parahaemolyticus* lacking *tdh*/*trh* genes were reported [47,48,49], and a study conducted in Italy showed that about 10% of the clinical strains related to infections contained neither the gene *tdh* nor *trh* [50]. Furthermore, in spite of the absence of *tdh* and *trh* genes, the *V. parahaemolyticus* strains can carry other markers of virulence and reveal high rates of resistance to antimicrobial drugs that could be ineffective for treating infections [10].

Referring to the assessment of antimicrobial resistance in *V. parahaemolyticus*, our findings are similar to those reported by other authors about the incidence of ampicillin resistance [10,31,33], while we found a lower rate of resistance to streptomycin compared to others [51]. Referring to cefazolin and cephalothin, our results are comparable to those reported by Shaw et al. in 2014 [33] and Kang et al. in 2017 [51], while greater rates of resistance were reported more recently [10,31]. Apart from slight differences in terms of percentages obtained, our findings confirmed that the *V. parahaemolyticus* isolates were resistant to ampicillin and first generation cephalosporins, in agreement with previous reports. This suggests that the first generation cephalosporins may have been misused widely, thereby reducing their efficiency in the treatment of *V. parahaemolyticus* infections [10,52].

Silva et al. [10] reported a 100% sensitivity for tetracycline and gentamicin that, together with cefotaxime, are included among the most efficient antibiotics against *Vibrio parahaemolyticus* [31]. On the contrary, our results showed that 14% and 4.6% of the isolates of *V. parahaemolyticus* were resistant to tetracycline and gentamicin, respectively, while intermediate resistance to cefotaxime was observed in 14% of them.

Our findings about *V. cholerae* confirm high rates of complete or intermediate resistance to antibiotics included in therapeutic protocols, such as ampicillin and streptomycin [18,31], and susceptibility to cefotaxime, ciprofloxacin, chloramphenicol, and tetracycline [18]. Our results confirm the sensitivity of *V. vulnificus* to antibiotics, including drugs recommended by the Centers for Disease Control and Prevention (CDC) for the treatment of infections—tetracycline, ciprofloxacin, and trimethoprim/sulfamethoxazole [53]—except for one strain of *V. vulnificus* that showed intermediate sensitivity to tetracycline and ciprofloxacin and resistance to cefazolin. Referring to *V. alginolyticus* isolates, our results are comparable to those of other authors that report more than an 80% incidence of resistance to beta-lactams and 100% susceptibility to ceftriaxone [42].

Some studies that were conducted to characterize the antibiotic susceptibility profile of *Vibrio* spp. indicated that *V. parahaemolyticus* and *V. vulnificus* have developed multiple antibiotic resistances, which may lead to the failure of the available treatment options for common infections [54]. Although ampicillin is widely accepted as a first choice drug in the treatment of foodborne diseases, it has a low efficacy against *Vibrio* spp. [55,56], because first generation antibiotics, including ampicillin, are widely used in aquaculture, thus reducing the sensitivity of *Vibrio* to these antibiotics and the effectiveness of treatment [31,57].

## 5. Conclusions

The present study highlighted the role of seafood and especially bivalves as potential sources of resistant *Vibrio* spp. that possibly are disseminated to humans after ingestion. About 30% of bivalves and 20% of other fishery products were contaminated by *Vibrio* spp.; even though the majority of isolates were non-pathogenic, high rates of antimicrobial resistance were observed especially for ampicillin. An alarming result is represented by the detection of strains resistant to tetracycline and gentamicin, currently included among the most efficient antibiotics against *Vibrio* spp. In spite of its low incidence, resistance to these antibiotics might pose further issues in treating infections, and their spread in the environment should be monitored. The detection of *Vibrio* spp. in molluscs collected at retail outlets highlights that current depuration treatments are ineffective, and new ones should be implemented, in order to guarantee the protection of consumers. Moreover, in order to avoid cross-contamination at retail, each type of seafood should be kept in separate areas with no reciprocal contact. Finally, even though thorough cooking might limit the risk of foodborne illness, potential cross-contamination during preparation or consumption of raw or undercooked seafood might pose a risk of *Vibrio* infection. For this reason both consumers and professionals in the catering sector should be properly informed and trained, in order to both increase their awareness of the issue and improve their management skills for adequate food preservation and preparation.

## Figures and Tables

**Table 1:** Primer sequences used in this study and size expected for the specific amplicons.

Target Species	Genetic Marker	Primer Sequences (5′-3′)	Product Size	References
*Vibrio parahaemolyticus*	*toxR*	F: GTCTTCTGACGCAATCGTTG R: ATACGAGTGGTTGCTGTCATG	368 bp	[37]
	*tdh*	F: GTAAAGGTCTCTGACTTTTGGAC R: TGGAATAGAACCTTCATCTTCAC	269 bp	[38]
	*trh*	F: TTGGCTTCGATATTTTCAGTATCT R: CATAACAAACATATGCCCATTTCCG	500 bp	[38]
*Vibrio vulnificus*	*VVH*	F: CCGGCGGTACAGCTTGGCGC R: CGCCACCCACTTTCGGGCC	519 bp	[39]
*Vibrio cholerae*	*prVC*	F: TTAAGCSTTTTCRCTGAGAATG R: AGTCACTTAACCATACAACCCG	295–310 bp	[39]
	*ctxA*	F: CGGGCAGATTCTAGACCTCCTG R: CGATGATCTTGGAGCATTCCCAC	564 bp	[40]
	*stn*/*sto*	F: TCGCATTTAGCCAAACAGTAGAAA R: GCTGGATTGCAACATATTTCGC	172 bp	[40]
	*tcpA*	F: CACGATAAGAAAACCGGTCAAGAG R: TTACCAAATGCAACGCCGAATG	620 bp	[40]
	*hlyAET*	F: GGCAAACAGCGAAACAAATACC R: CTCAGCGGGCTAATACGGTTTA	481 bp	[40]

**Table 2:** Antimicrobial resistances of *Vibrio* spp. strains analyzed in this study.

Drugs	Strains											
	*V. parahaemolyticus*			*V. vulnificus*			*V. cholerae* NCV			*V. alginolyticus*		
	n = 43			n = 5			n = 12			n = 105		
	R ^a^	I ^b^	MDR ^c^	R	I	MDR	R	I	MDR	R	I	MDR
**Amp**	30		2 ^d^				9			93		
**Ctx**		6										
**Cro**							2					
**Caz**		8					2	2		12	13	
**Kf**	9	9					2	6				
**Cip**					1							
**C**												
**Cn**	2									23	12	
**K**	2	8									11	
**S**	8	9		5				2			58	
**Stx**							5					
**Te**	6	4			1					12		
**Ct**	2	9										
**Kz**	13			1								
**Amc**	2	2										

^Amp^ Ampicillin, 10 μg. ^Ctx^ Cefotaxime, 30 μg. ^CRO^ Ceftriaxone, 30 µg. ^Caz^ Ceftazidime, 10 μg. ^Kf^ Cephalothin, 30 μg. ^Cip^ Ciprofloxacin, 5 μg, ^C^ Chloramphenicol, 10 μg. ^Cn^ Gentamicin, 10 μg. ^K^ Kanamycin, 30 μg. ^S^ Streptomycin, 10 μg. ^Stx^ Trimethoprim/Sulfamethoxazole, 25 μg. ^Te^ Tetracycline, 10 μg. ^Ct^ Colistin Sulphate, 10 μg. ^Kz^ Cefazolin, 30 µg. ^Amc^ Amoxicillin/Clavulanic Acid, 20 μg/10 μg. ^a^ Resistant. ^b^ Intermediate. ^c^ Multidrug resistant. ^d^ MDR pattern AMP/KF/S.

## Data Availability

Not applicable.
